# Covalent adduct formation between the plasmalogen-derived modification product 2-chlorohexadecanal and phloretin

**DOI:** 10.1016/j.bcp.2014.12.017

**Published:** 2015-02-15

**Authors:** Andreas Üllen, Christoph Nusshold, Toma Glasnov, Robert Saf, David Cantillo, Gerald Eibinger, Helga Reicher, Günter Fauler, Eva Bernhart, Seth Hallstrom, Nora Kogelnik, Klaus Zangger, C. Oliver Kappe, Ernst Malle, Wolfgang Sattler

**Affiliations:** a Institute of Molecular Biology and Biochemistry, Medical University of Graz, Graz, Austria; b Christian Doppler Laboratory for Flow Chemistry, Institute of Chemistry, University of Graz, Graz, Austria; c Institute of Chemistry and Technology of Materials, Graz University of Technology, Graz, Austria; d Institute of Chemistry, University of Graz, Graz, Austria; e Clinical Institute of Medical and Chemical Laboratory Diagnostics, Medical University of Graz, Graz, Austria; f Institute of Physiological Chemistry, Medical University of Graz, Graz, Austria

**Keywords:** Chlorinated fatty aldehyde, Blood–brain barrier, Neuroinflammation, Myeloperoxidase, Plasmalogens

## Abstract

Hypochlorous acid added as reagent or generated by the myeloperoxidase (MPO)-H_2_O_2_-Cl^−^ system oxidatively modifies brain ether-phospholipids (plasmalogens). This reaction generates a sn2-acyl-lysophospholipid and chlorinated fatty aldehydes. 2-Chlorohexadecanal (2-ClHDA), a prototypic member of chlorinated long-chain fatty aldehydes, has potent neurotoxic potential by inflicting blood–brain barrier (BBB) damage. During earlier studies we could show that the dihydrochalcone-type polyphenol phloretin attenuated 2-ClHDA-induced BBB dysfunction. To clarify the underlying mechanism(s) we now investigated the possibility of covalent adduct formation between 2-ClHDA and phloretin. Coincubation of 2-ClHDA and phloretin in phosphatidylcholine liposomes revealed a half-life of 2-ClHDA of approx. 120 min, decaying at a rate of 5.9 × 10^−3^ min^−1^. NMR studies and enthalpy calculations suggested that 2-ClHDA-phloretin adduct formation occurs via electrophilic aromatic substitution followed by hemiacetal formation on the A-ring of phloretin. Adduct characterization by high-resolution mass spectroscopy confirmed these results. In contrast to 2-ClHDA, the covalent 2-ClHDA-phloretin adduct was without adverse effects on MTT reduction (an indicator for metabolic activity), cellular adenine nucleotide content, and barrier function of brain microvascular endothelial cells (BMVEC). Of note, 2-ClHDA-phloretin adduct formation was also observed in BMVEC cultures. Intraperitoneal application and subsequent GC–MS analysis of brain lipid extracts revealed that phloretin is able to penetrate the BBB of C57BL/6J mice. Data of the present study indicate that phloretin scavenges 2-ClHDA, thereby attenuating 2-ClHDA-mediated brain endothelial cell dysfunction. We here identify a detoxification pathway for a prototypic chlorinated fatty aldehyde (generated via the MPO axis) that compromises BBB function in vitro and in vivo.

## Graphical abstract

**Figure:**
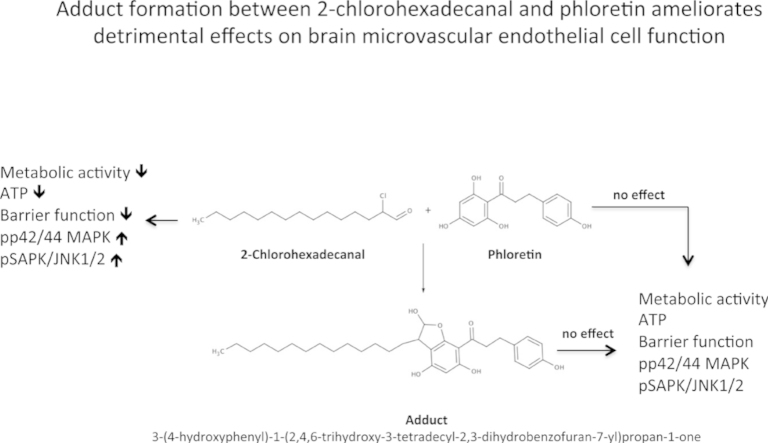

## 1 Introduction

The neurovascular unit physically separates most regions of the brain from the periphery to maintain central nervous system homeostasis [1]. Within this specialized vessel system, brain microvascular endothelial cells (BMVEC) constitute the morphological basis of the blood–brain barrier (BBB) [2]. The formation of tight junctions prevents paracellular transport of molecules and cells and maintains homeostasis of the brain micromilieu via elaborately regulated transport mechanisms.

Under inflammatory conditions BBB function is compromised and can aggravate neuronal dysfunction [3]. Many pathways that compromise BBB and neuronal function in further consequence have been shown to converge on the formation of reactive species [4]. This is of particular importance since tight junction proteins are sensitive to alterations of the intracellular redox status, ultimately resulting in barrier dysfunction [5]. Various effects of reactive oxygen species e.g. inhibition of cerebral blood flow and alterations in barrier integrity have been demonstrated in cerebrovascular diseases and stroke [6–8]. Edema formation in stroke causes an increase in cell volume, depolarization, breakdown of ionic gradients, cellular ATP depletion, and finally vasogenic edema in response to BBB breakdown [8].

During our earlier studies we could show pronounced BMVEC barrier dysfunction in response to the fatty aldehyde 2-chlorohexadecanal (2-ClHDA) that is generated during endotoxemia [9,10]. 2-ClHDA is formed by attack of plasmalogens (ether phospholipids) by hypochlorous acid (HOCl) [11,12]. HOCl in turn is generated by the myeloperoxidase (MPO)–H_2_O_2_–Cl^−^ system of activated phagocytes [13], cells that release MPO at the cerebrovasculature [10]. Under physiological conditions MPO is part of the innate immune system, however, under chronic inflammatory conditions the MPO–H_2_O_2_–Cl^−^ system is implicated in the development of (neurological) diseases. MPO is abundantly expressed in microglia in and around demyelinated lesions in Multiple Sclerosis in humans and rodents [14]. In line, pharmacological inhibition of MPO reduced the severity of clinical symptoms in a murine model of Multiple Sclerosis [15]. The involvement of MPO in barrier dysfunction was also demonstrated during bacterial meningitis [16,17]. We recently reported significantly elevated MPO levels in mouse brain in response to systemic lipopolysaccharide (LPS) administration [9]. MPO expression was accompanied by a significant decrease of brain plasmalogen content and concomitant formation of 2-ClHDA [9]. In line with deleterious effects of MPO-generated 2-ClHDA we could show that LPS-induced BBB dysfunction was significantly less pronounced in MPO^−/−^ mice as compared to the corresponding littermates [10].

Zhu and colleagues [18] suggested that chemical trapping of reactive aldehydes by polyphenols could provide an attractive approach to prevent cellular dysfunction in response to Schiff's base formation between vital cellular proteins and reactive aldehydes. We recently reported that phloretin, a dihydrochalcone-type polyphenol, prevented 2-ClHDA-induced barrier dysfunction, apoptosis, and ATP depletion in BMVEC in vitro by yet unidentified mechanisms [19]. To reveal the underlying mechanisms of adduct formation between 2-ClHDA and phloretin we applied NMR analysis, theoretical enthalpy calculations, and high-resolution mass spectroscopy (HRMS). To get an indication about altered toxicity profiles after adduct formation, metabolic activity, the cellular adenine nucleotide status, and barrier function was studied in BMVEC. To obtain first evidence about BBB permeability of phloretin in vivo, uptake of i.p. injected phloretin was quantitated in murine brain.

## 2 Materials and methods

Cell culture supplies were from Gibco (Vienna, Austria), PAA Laboratories (Linz, Austria), Costar (Vienna), or VWR (Vienna). Dulbecco's modified Eagle's medium (DMEM) Ham's F12, hydrocortisone, *N*-chlorosuccinimide, dl-proline, dipalmitoylphosphatidylcholine (DPPC), phloretin (3-(4-hydroxyphenyl)-1-(2,4,6-trihydroxyphenyl) propan-1-on), resveratrol, pentafluorobenzyl (PFB) hydroxylamine, dimethyl sulfoxide (DMSO), and 3-(4,5-dimethyl-2-thiazolyl)-2,5-diphenyltetrazolium bromide (MTT) were from Sigma-Aldrich (Vienna). Electrical cell-substrate impedance sensing (ECIS) electrode arrays (8W10E+) were from Ibidi (Martinsried, Germany). Hexadecanal was from Toronto Research Chemicals (Toronto, Canada). *N*-Methyl-*N*-(trimethylsilyl)-trifluoroacetamide (MSTFA) was from ABCR (Karlsruhe, Germany), trimethylchlorosilane (TMCS) and pyridine were from Pierce (Rockford, IL, USA). Silica 60 gel and silica 60 TLC plates were from Merck (Darmstadt, Germany). Poly-Prep® chromatography columns were from Bio-Rad (Vienna). Phosphospecific rabbit polyclonal anti-pp44/42 MAPK (Thr202/Tyr204), anti-pSAPK/JNK1/2 (Thr183/Tyr185), pan-specific rabbit monoclonal anti-p44/42, and rabbit polyclonal anti-SAPK/JNK1/2 antibodies were from Cell Signaling Technology (Beverly, MA). HRP-labeled secondary goat anti-rabbit IgG was from Pierce (Rockford, IL). All solvents and other reagents of analytical grade were from Merck, Sigma-Aldrich, or Roth.

### 2.1 Synthesis of 2-chlorohexadecanal (2-ClHDA)

2-ClHDA was synthesized by organocatalytic α-chlorination of hexadecanal [20]. dl-Proline (0.2 equ.) followed by *N*-chlorosuccinimide (1.5 equ.) were added to a solution of hexadecanal (1 equ.) in 4 ml CH_2_Cl_2_ at 0 °C. The suspension was allowed to warm to ambient temperature and was stirred over night. Hexane (15 ml) was added to the reaction mixture and the precipitate was filtered. 2-ClHDA was purified using a Silica 60 column and hexane/diethyl ether (90:10, v/v) as eluent (yield = 51%). 2-Cl[^13^C_8_]HDA used as internal standard for gas chromatography–mass spectroscopy (GC–MS) analysis was synthesized and purified as described [9].

### 2.2 Analytical procedures

DPPC liposomes containing equimolar concentrations of phloretin and 2-ClHDA were prepared by dispersing a mixture of 2.5 mg DPPC (0.7 mM), 350 μg phloretin (0.3 mM), and 350 μg 2-ClHDA (0.3 mM) in 2 ml phosphate-buffered saline (PBS, pH 7.4). The mixture was sonicated on ice for 3 × 10 s. The reaction was started by gently stirring the mixture at 37 °C in the dark under an argon atmosphere. At the indicated time points 100 μl aliquots (approx. 18 nmol of reactants) were removed and 250 ng 2-Cl[^13^C_8_]HDA [9] was added. Samples were immediately extracted twice with ethyl acetate (2 ml) followed by a Folch extraction. Extracts were stored at −70 °C until analysis. After conversion to the corresponding PFB-oxime derivatives 2-ClHDA was quantitated by negative ion chemical ionization (NICI)–GC–MS. Alternatively adduct formation was studied in acetonitrile, tetrahydrofurane (THF; both solvents containing 1% triethylamine; 240 μM 2-ClHDA and phloretin), or high-density lipoproteins (HDL; 80 μM 2-ClHDA and phloretin in PBS containing 250 μg HDL protein/ml). HDL was isolated from plasma as described [21]. Following extraction, phloretin and adduct concentrations were quantitated by HPLC (see below). A one-phase exponential decay model (*C*_*t*_ = *C*_0_ × e^−*kt*^) was used to fit experimental data using the Prism 5.0 software (GraphPad, San Diego, CA, USA).

### 2.3 Derivatization for GC–MS

Preparation of PFB-oxime derivatives of 2-ClHDA and 2-Cl[^13^C_8_]HDA for NICI–GC–MS was performed as described above. Phloretin and resveratrol were converted to the corresponding trimethylsilyl (TMS)-ether derivatives in 100 μl MSTFA/pyridine (2:1; v/v) containing 1% (v/v) TMCS at room temperature (RT) for 30 min immediately before electron impact (EI)–GC–MS analysis due to putative phloretin keto-enol tautomerism.

### 2.4 NICI–GC–MS and EI–GC–MS

Samples were separated on a Thermo Scientific Trace GC Ultra (helium was used as carrier gas, 2 ml/min) with a Zebron ZB-AAA capillary column (15 m, 0.25 mm inner diameter, Phenomenex^®^) and analyzed using a DSQII mass spectrometer (Thermo Scientific).

For NICI–GC–MS the injector temperature was set to 280 °C. The oven temperature was maintained at 100 °C for 5 min, increased during the first ramping step at a rate of 20 °C/min to 175 °C, and held at 175 °C for 5 min. In the second ramping step the temperature was raised at a rate of 15 °C/min to 280 °C and held at 280 °C for additional 2 min. The transfer line was kept at 300 °C and the ion source temperature was 100 °C. All spectra were monitored in the NICI mode (methane was used as reagent gas), either in full scan or using selected ion monitoring (SIM) mode. In SIM, target compounds were identified at the molecule-specific mass-to-charge ratios 288/290 (2-ClHDA) or 296/298 (2-Cl[^13^C_8_]HDA) and by the characteristic isotope distribution of chlorine (^35^Cl/^37^Cl, 3:1). Quantitation of 2-ClHDA was performed by peak area comparison with 2-Cl[^13^C_8_]HDA.

For EI–GC–MS the injector temperature was set to 230 °C. The oven temperature was maintained at 100 °C for 3 min, increased during the first ramping step at a rate of 30 °C/min to 280 °C, and held at 280 °C for 2 min. In the second ramping step the temperature was raised at a rate of 30 °C/min to 320 °C. The transfer line was kept at 320 °C and the ion source temperature was 200 °C. Mass spectra were recorded either in full scan or SIM mode with an electron ionization energy of 70 eV and an emission current of 100 μA. In SIM, the diagnostic ions used for four-fold silylated phloretin were at *m*/*z* 547 and *m*/*z* 562. Three-fold silylated resveratrol was detected at *m*/*z* 429 and *m*/*z* 444. Quantitation of phloretin was performed by peak area comparison using resveratrol as internal standard.

### 2.5 Isolation and characterization of 2-ClHDA-conjugated phloretin

DPPC-liposomes containing phloretin and 2-ClHDA were prepared as described above. After 16 h samples were extracted twice with ethyl acetate followed by the Folch procedure. Subsequently, extracts were separated by TLC on silica 60 plates and CHCl_3_/MeOH (8:1, v/v) as mobile phase. Under these conditions phloretin migrated with an *R*_*f*_ of 0.42 and the reaction product at an *R*_*f*_ of 0.61. Bands were visualized by spraying plates with 2% (w/v) FeCl_3_ in 0.5 M HCl and subsequent heating (≈90 °C, 3 min). For isolation of 2-ClHDA-conjugated phloretin DPPC-liposomes (15 mg DPPC, 0.9 mM) containing 1.5 mg phloretin (0.2 mM) and 1.7 mg 2-ClHDA (0.3 mM) in PBS (pH 7.4) were gently stirred for 24 h at 37 °C under argon in the dark. Extraction was performed twice with ethyl acetate followed by the Folch procedure. Combined organic layers were dried in vacuum and conjugated phloretin was pre-purified using a Silica 60 column and CHCl_3_/MeOH (8:1, v/v) as eluent, followed by preparative TLC using CHCl_3_/MeOH (8:1, v/v) as mobile phase. A second lane (used as reference) was cut-off the plate and stained with FeCl_3_ as described above. Comigrating bands were scraped off, extracted with CHCl_3_/MeOH and dried under N_2_. The fraction of the newly formed main reaction product (*R*_*f*_ 0.61) was scrapped off, extracted twice from the TLC sorbent with CHCl_3_/MeOH (2:1, v/v) and analyzed by HRMS (see below).

### 2.6 HPLC analysis

Homogeneity of the isolated fractions was analyzed by reversed-phase HPLC on a Waters HPLC 2690 Module and a Waters UV 2487 detector (set at 288 nm). Separation was carried out on a Kromasil C18 column (150 × 4.6 mm, Altmann Analytik, Munich, Germany) at a flow rate of 0.8 ml/min by isocratic elution using acetonitrile/water (5%, v/v) as mobile phase. Under these chromatographic conditions baseline separation is achieved (retention time of phloretin and adduct = 1.9 and 7.7 min, respectively). Quantitation was performed with external calibration curves.

### 2.7 NMR

Adduct formation was performed in DPPC liposomes as described above. For the complete assignment of proton and carbon signals, 1D (^1^H, homodecoupled ^1^H [22], ^13^C and DEPT-135) as well as 2D (COSY, HSQC, HMBC, and INADEQUATE) spectra were acquired. The INADEQUATE experiment was recorded on a Bruker Avance III 700 MHz NMR spectrometer using a 5 mm TCI cryogenically cooled probe. All other spectra were obtained on a Bruker Avance III 500 MHz NMR spectrometer at 298 K using DMSO-d_6_ as the solvent. The spectra were processed and analyzed using TopSpin 3.1 and MestReNova 8.0. Chemical shifts were referenced relative to the residual solvent signal.

### 2.8 Enthalpy calculations

All of the calculations were carried out using the Gaussian 09 package [23]. The B3LYP density-functional method [24] in conjunction with the 6-311G(d,p) basis set was selected for all the geometry optimizations and frequency analysis. The geometries were optimized including solvation effects. For this purpose, the SMD solvation method [25] was employed using water as a solvent. Frequency calculations at 298.15 K on all the stationary points were carried out at the same level of theory as the geometry optimizations to ascertain the nature of the stationary points. Ground and transition states were characterized by none and one imaginary frequency, respectively. All of the presented relative energies are free energies at 298.15 K. To simplify the calculations a model of the reactants with *R* and *R*′ = CH_3_ was used (see Fig. 3).

### 2.9 High-resolution mass spectrometry (HRMS)

Adduct formation was performed in DPPC liposomes as described above. Matrix-assisted laser desorption ionization-time of flight (MALDI-TOF) mass spectrometry was performed on a Micromass TofSpec 2E time-of-flight mass spectrometer. Ions were generated by irradiation just above the threshold laser power (laser: wavelength 337 nm, operated at a frequency of 5 Hz). Positive ion spectra were recorded in reflectron mode applying an accelerating voltage of 20 kV and externally calibrated with a suitable mixture of polyethyleneglycols. The spectra of 100–150 shots were averaged. Analysis of data was done with MassLynx-Software V4.1 (Micromass/Waters, Manchester, UK). Samples were prepared by mixing a solution of dithranol (10 mg/ml in THF), a solution of the analyte (obtained from TLC as described above), and a solution of F_3_CCOONa (NaTFA; 0.1 mg/ml in THF) in the cap of a microtube in a ratio of 10:2:1 (v/v/v). Samples (0.5 μl) were deposited on the plate (stainless steel) and allowed to dry under air.

High-resolution EI mass spectra (70 eV, source temperature 250 °C) were recorded on an orthogonal TOF spectrometer (Waters GCT Premier) equipped with a direct insertion (DI) probe. 0.5 μl of a solution of the sample that was obtained from TLC were placed in the glass cup used for DI, dried under atmospheric pressure, and transferred into the vacuum. The acquisition of mass spectra (mass range: 50–800 Da; 1 spectrum/s; resolution: approx. 7500 FWHM) was started immediately. Spectra were continuously acquired while the sample was heated from RT to 400 °C (heating rate: 40 °C/min). Data were processed using MassLynx (version 4.1).

### 2.10 Isolation and culture of primary porcine BMVEC

BMVEC from porcine brains were isolated by a combination of mechanical disintegration, enzymatic digestion, and centrifugation steps and were cultured exactly as described [10].

For cell culture experiments 2-ClHDA, phloretin, and the 2-ClHDA-phloretin adduct were prepared as stock solutions in DMSO. Final DMSO concentrations in culture medium were 0.2% (v/v; impedance measurements) or 0.4% (v/v; MTT test and adduct formation in BMVEC cultures).

### 2.11 MTT test

To investigate the effects of 2-ClHDA-conjugated phloretin on metabolic activity the MTT test was used [19]. Briefly, BMVEC were grown in 96-well plates to confluence, serum starved over night, and treated with 2-ClHDA, phloretin, or the 2-ClHDA-phloretin adduct in serum-free medium at the indicated concentrations for 5 h. After treatment, the medium was replaced by serum-free medium (100 μl per well) containing MTT (1.2 mM) and the cells were incubated for 1 h under standard conditions. The cells were washed (PBS) and lysed (isopropanol/1 M HCl, 24:1 (v/v); 100 μl/well) on a rotary shaker (1000 rpm, 15 min). Absorbance was measured at 570 nm on a Victor multilabel reader (Wallac) and corrected for background absorption (650 nm).

### 2.12 Adenine nucleotide analysis

BMVEC were cultured to confluence in 6-well plates and serum-starved for 24 h before phloretin, 2-ClHDA, phloretin plus 2-ClHDA, or the 2-ClHDA-phloretin adduct (25 μM each) was added in serum-free medium. After 8 h the cells were washed, trypsinized and cellular proteins were precipitated with 0.4 M perchloric acid. After centrifugation (12,000 × *g*), 100μl of the supernatant were neutralized with 20–25 μl of potassium carbonate (2 M, 4 °C). The supernatant (40 μl) obtained after centrifugation was used for HPLC analysis as described [26]. The pellets of the acidic extract were dissolved in 50 μl of NaOH (0.1M) and used for protein determination at a dilution of 1:10 (BCA Assay; Pierce).

### 2.13 Electric cell-substrate impedance sensing (ECIS)

BMVEC barrier function was quantitated by impedance measurement at 4 kHz on collagen-coated gold electrodes of 8W10E+ arrays using an ECIS Z System (Applied Biophysics, Troy, NY, USA). After induction of tight junction formation by hydrocortisone endothelial cells were challenged with 2-ClHDA, phloretin, or the 2-ClHDA-phloretin adduct.

### 2.14 Western blot analysis

BMVEC protein lysates obtained from cells that were time-dependently incubated with 2-ClHDA (15 μM; in the absence or presence of phloretin, 15 μM) were separated by SDS-PAGE and electrophoretically transferred to PVDF membranes. Phosphospecific antibodies (rabbit) against pp44/42 and, pSAPK/JNK1/2 (diluted 1:500; 3% BSA in TBS) were applied by overnight incubation at 4 °C. Immunoreactive bands were visualized using HRP-conjugated goat anti-rabbit IgG (1:5000 in 5% (w/v) skim milk powder in TBS, 2 h, RT) and subsequent ECL Plus development. For normalization, membranes were stripped at 50 °C for 30 min with gentle shaking and reprobed with primary antibodies against corresponding pan-proteins (overnight at 4 °C, diluted 1:1000, 5% (w/v) skim milk powder in TBS). Detection of immunoreactive bands was performed as mentioned above with HRP-conjugated goat anti-rabbit or goat anti-mouse secondary antibodies (1:5000 in 5% (w/v) skim milk powder in TBS, 2 h, RT) using a Bio Rad ChemiDoc MP system.

### 2.15 Adduct formation in BMVEC cultures

BMVEC were grown in collagen-coated 6-well plates to confluence and were preincubated in the presence of phloretin in glucose-containing HBSS buffer (pH = 7) for 60 min at 37 °C before addition of 2-ClHDA (25 μM, final concentration). For adduct analysis 1 ml of the supernatant was extracted twice with 2 ml ethyl acetate and processed for HPLC analysis (see below). For 2-ClHDA analyses 1 ml medium (containing 200 ng 2-Cl[^13^C_8_]HDA as internal standard) was extracted twice with 2 ml hexane/methanol (5:1, v/v). Cells were washed with PBS and cellular lipids were extracted in the presence of 200 ng 2-Cl[^13^C_8_]HDA using two consecutive extractions (30 min, RT) with 1 ml hexane/isopropanol (3:2, v/v) on a rotary shaker (1000 rpm). 2-ClHDA-conjugated phloretin was quantitated by reversed-phase HPLC and 2-ClHDA concentrations were quantitated using NICI–GC–MS after preparation of PFB-oxime derivatives*.*

### 2.16 Animals and animal experiments

Male C57BL/6J mice (8–10 weeks, 20–30 g) were obtained from the ‘Institut für Versuchstierkunde’ (Himberg, Austria). All animal experiments were performed in accordance with animal care ethics approval and guidelines, as per Animal Care Certificate BMWF-66.010/0055-II/3b/2011 of the Austrian Federal Ministry of Science and Research (Vienna). All animals were kept on a 12 h light/dark cycle with free access to food and water. Mice received a single i.p. dose of phloretin (0.1 mg/g body weight, 100 mM in DMSO). After 15, 30, 60, 120, and 240 min mice were anesthetized with 150 mg/kg pentobarbital (Nembutal) and transcardially perfused with 25 ml PBS. Subsequently, brains were removed, weighed, snap-frozen in liquid N_2_, and stored at −70 °C until further processing. Frozen brain tissues were homogenized using a BioPulverizer (BioSpec Products, Bartlesville, USA) and extracted twice with ethyl acetate in the presence of 1 μg resveratrol (internal standard), which was followed by a Folch extraction. Polyphenols were purified from lipid extracts by solid phase extraction using silica 60 columns, using CHCl_3_/MeOH (8:1, v/v) as eluent. After conversion to TMS-ether derivatives (as described above) polyphenols were quantified by EI–GC–MS.

### 2.17 Statistical analyses

Data are presented as means ± SD. To test differences in groups, statistical significance was determined by one-way ANOVA with Bonferroni correction (using the GraphPad 5.0 Prism package) as indicated. All values of *p* ≤ 0.05 were considered significant. * *p* < 0.05, ** *p* < 0.01, *** *p* < 0.001; * compared to vehicle; # compared to the same concentration of 2-ClHDA.

## 3 Results

### 3.1 Time-dependent scavenging of 2-ClHDA by phloretin

**Fig. 1:**
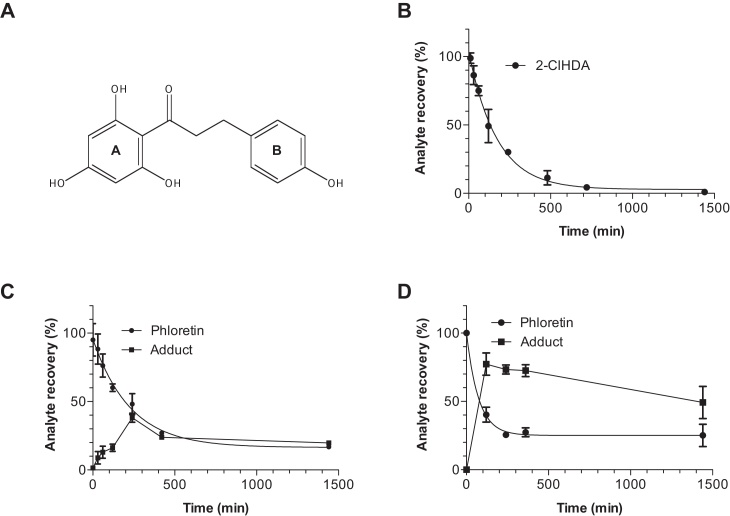
**Analyte recovery during 2-ClHDA-phloretin adduct formation.** (A) Phloretin structure. The A- and B-ring is indicated. (B) DPPC liposomes containing 2-ClHDA and phloretin were incubated at 37 °C in PBS. (C) 2-ClHDA and phloretin were incubated in acetonitrile (containing 0.1% triethylamine) at 37 °C. (D) 2-ClHDA and phloretin were incubated in PBS containing 250 μg HDL protein/ml at 37 °C. At the indicated time points aliquots of the reaction mixture were extracted, and either converted to the corresponding PFB-oxime derivatives and analyzed by NICI–GC–MS using 2-Cl[^13^C_8_]HDA as internal standard (B) or were quantitated by HPLC analysis (C and D) using external calibration. Results are presented as percentage recovery of the corresponding analytes. Results represent mean ± SD from triplicate experiments.

To monitor the rate of 2-ClHDA scavenging by phloretin (structure shown in Fig. 1A) the reactants were incorporated in DPPC liposomes at equimolar ratios. At the indicated times aliquots of the reaction mixture were removed, extracted, derivatized, and analyzed by NICI–GC–MS. These analyses revealed that 2-ClHDA concentrations decreased time-dependently (Fig. 1B) which could be fitted according to a first order decay (*C*_*t*_ = *C*_*o*_ × *e*^−*kt*^; *r*^2^ = 0.98). Of note, the constants reported here are observed rate constants, not second order rate constants. Under the experimental conditions used the half-life (*τ*/2) of 2-ClHDA was 120 min, with a calculated decay rate of 5.9 × 10^−3^ min^−1^. At the latest time point (24 h) virtually all 2-ClHDA was consumed.

Taking into consideration that both compounds are lipophilic and could accumulate in hydrophobic compartments (logP values: phloretin > 2, Ref. [27]; 2-ClHDA = 6.2, ChemDraw calculation) of biological systems we have analyzed adduct formation in acetonitrile or THF (both solvents containing 1% triethylamine). While adduct formation in THF was only observed at 50 °C (data not shown), time-dependent loss of phloretin in acetonitrile revealed a decay (4.4 × 10^−3^ min^−1^) and a *τ*/2 (156 min; Fig. 1C) comparable to what was observed in liposome preparations. During these experiments adduct accumulation did not match loss of phloretin (Fig. 1C). Formation of a disubstituted phloretin adduct in analogy to what was reported for acrolein scavenging by phloretin [18] could provide a possible explanation for this observation. This issue was, however, not further addressed experimentally during the present study. Since BMVEC actively secrete lipoproteins of the high-density range [28] we have evaluated whether HDL could provide a hydrophobic environment that facilitates 2-ClHDA-phloretin adduct formation. These experiments revealed a decay rate for phloretin of 1.4 × 10^−2^ min^−1^ and a *τ*/2 of 51 min (Fig. 1D) indicating most efficient adduct formation under these conditions.

### 3.2 NMR structure elucidation

**Fig. 2:**
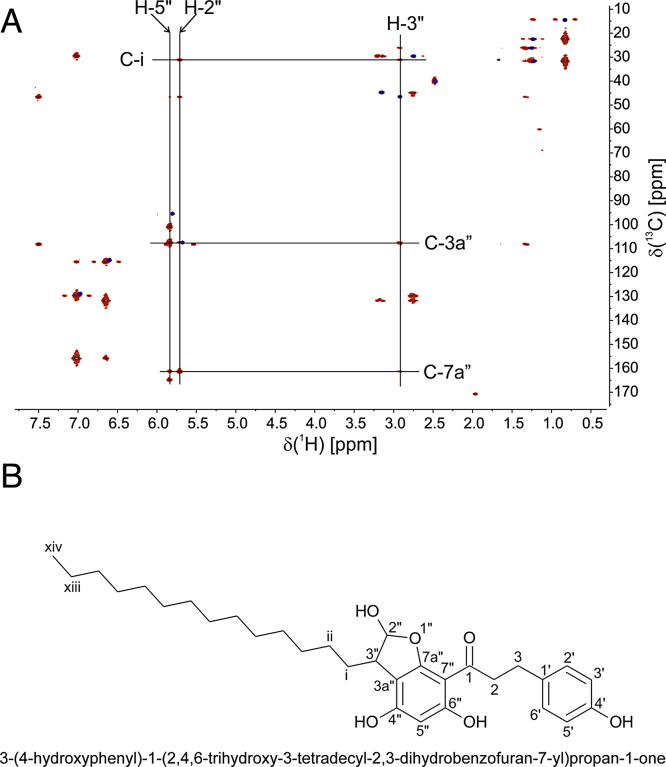
**NMR analysis of the 2-ClHDA-phloretin adduct.** (A) Overlay of the HSQC (blue) and HMBC (red) spectra of the 2-ClHDA-phloretin adduct. Key connectivities are indicated. (B) Adduct structure, name, and numbering scheme as applied in A and Table 1.

Hypothetically adduct formation between 2-ClHDA and phloretin could occur via electrophilic aromatic substitution followed by hemiacetal formation on the A-ring. To characterize the structure of the 2-ClHDA-phloretin adduct, NMR analyses and theoretical enthalpy calculations were performed. The isolated product was dissolved in DMSO-d_6_ and could be completely assigned using a combination of 1D (^1^H, homodecoupled ^1^H, ^13^C, DEPT-135) and 2D (COSY, HSQC, HMBC, INADEQUATE) spectra. Both the HMBC and INADEQUATE spectra were necessary to unambiguously identify the adduct structure. An overlay of the HSQC and HMBC spectra, confirming hemiacetal formation is shown in Fig. 2. In particular, correlations between H-2″ with C-7a″, H-3″ with C-3a″, and H-2″ with C-3a″ in the HMBC, as well as C-3″ with C-3a″ and C-3a″ with C-7a″ in the INADEQUATE were important for structure elucidation. The complete assignment is presented in Table 1. This analysis clearly suggests hemiacetal formation between the aldehyde and the A-ring of phloretin corresponding to the structure shown in Fig. 2B.

### 3.3 Enthalpy calculations

**Fig. 3:**
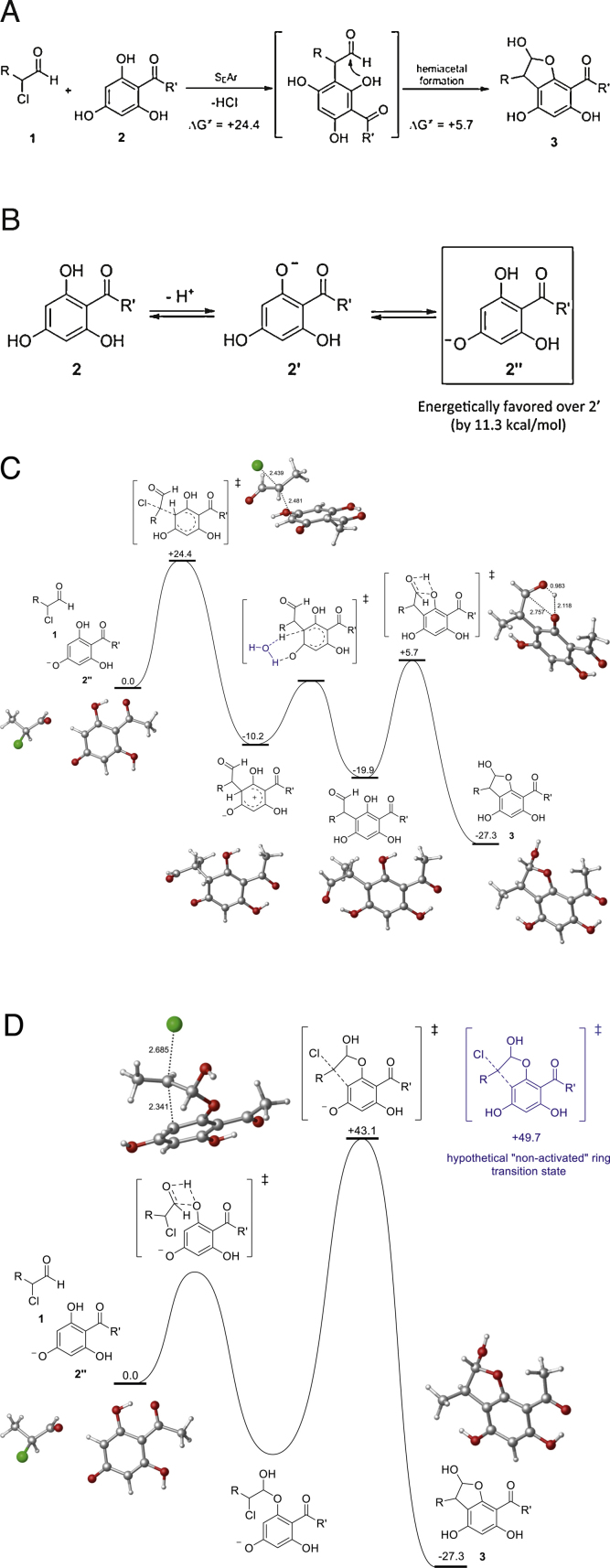
**Theoretical enthalpy calculations based on NMR data.** (A) Overall energy profile for the formation of the 2-ClHDA-phloretin adduct via electrophilic aromatic substitution followed by hemiacetal formation (*Pathway A*; 1 = 2-ClHDA, 2 = phloretin (for structure see Fig. 1A), 3 = adduct (for complete structure see Fig. 2B). To simplify enthalpy calculations *R* and *R*′ = CH_3_. (B) Under slightly basic conditions the A-ring of phloretin (2) is dissociated (2′ and 2″) and thus activated for electrophilic aromatic substitution. 2″ is more stable than 2′ (11.3 kcal/mole). (C) Energy profile (upper panel) for the formation of the 2-ClHDA-phloretin adduct via electrophilic aromatic substitution followed by hemiacetal formation (*Pathway* A). Relative free energy (in kcal/mol) with respect to 1 and 2″ are calculated at the B3LYP/6-311G(d,p) level. The ball and stick model (lower panel) shows optimized geometry of the key transition states involved in the formation of the 2-ClHDA-phloretin adduct via *Pathway A* (calculated distances are given in Å). (D) Energy profile (upper panel) for the formation of the 2-ClHDA-phloretin adduct via hemiacetal formation and subsequent electrophilic aromatic substitution (*Pathway B*). Relative free energy with respect to 1 and 2″ (in kcal/mol) are calculated at the B3LYP/6-311G(d,p) level. The ball and stick model (lower panel) shows optimized geometry of the key transition state involved in the formation of the 2-ClHDA-phloretin adduct via *Pathway B* (calculated distances are given in Å).

To further support these findings we have performed theoretical enthalpy calculations. These calculations imply that the formation of the 2-ClHDA-phloretin adduct is a two-step process which consists of an electrophilic aromatic substitution followed by a hemiacetal condensation on the A-ring of phloretin (Fig. 3A). To confirm this hypothesis and discard an alternative pathway where the formation of the hemiacetal occurs prior to dechlorination we performed a computational analysis of the reaction mechanism using density-functional theory calculations. In a slightly basic aqueous medium phloretin is dissociated and thus activated for electrophilic aromatic substitution (Fig. 3B). The theoretical energy barrier for the initial step is +24.4 kcal/mol, which is compatible with a process taking place after several hours at 37 °C. The ensuing open-intermediate rapidly evolves to the final 2-ClHDA-phloretin adduct through the formation of the hemiacetal moiety. The overall process is exothermic and 27.3 kcal/mol are released during adduct formation (Fig. 3C; *Pathway A*). The alternative pathway (Fig. 3D; *Pathway B*) where hemiacetal formation starts prior to chlorine abstraction represents a higher energy barrier (+43.1 kcal/mol) and is, therefore, less likely to occur. The optimized geometry of the key transition states involved in adduct formation via both pathways is displayed as ball-and-stick models in Fig. 3C and D, respectively. The calculated (ChemDraw) logP value of this adduct is 7.3.

### 3.4 HRMS characterization of the 2-ClHDA-phloretin adduct

**Fig. 4:**
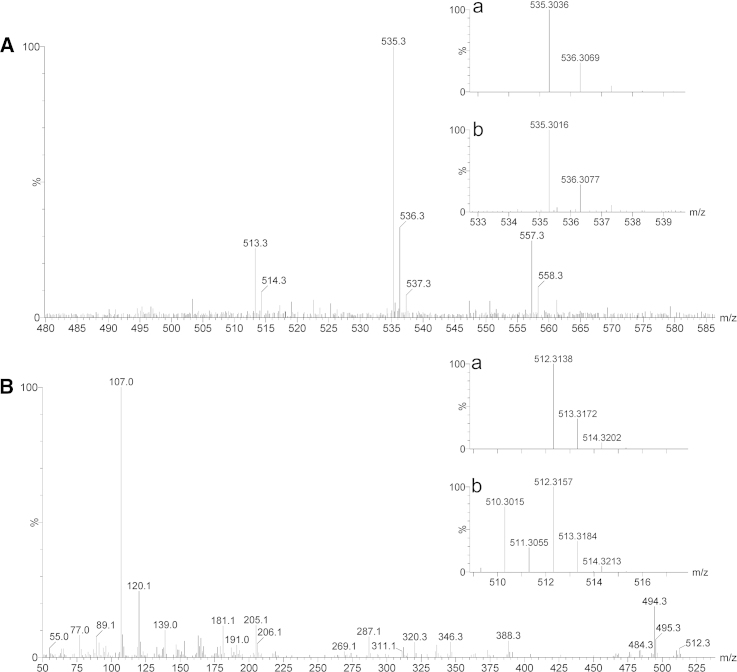
**HRMS analysis of the product obtained by reaction of 2-ClHDA with phloretin.** (A) MALDI-TOF mass spectrum of the 2-ClHDA-phloretin adduct. The insets display the calculated isotope pattern of [C_31_H_44_O_6_Na]^+^ (a), and the accurate mass data obtained experimentally (b). (B) Direct inlet-EI mass spectrum of the 2-ClHDA-phloretin adduct. The insets display the calculated isotope pattern of [C_31_H_44_O_6_]^+^ (a), and the accurate mass data obtained experimentally (b).

After reaction in liposomes, the adduct was isolated by TLC as described in the Methods section. One new major band (*R*_*f*_  = 0.61) was well separated from the original reactants. This band was scrapped off, extracted with CHCl_3_/MeOH (8:1, v/v), and subjected to HRMS analysis. The important part of the MALDI-TOF mass spectrum is shown in Fig. 4A. Three significant peaks were detected at *m*/*z* 513.3, 535.3, and 557.3 Da. These peaks are assigned to ions [MH]^+^, [MNa]^+^, and [(M − H + Na)Na]^+^, respectively, where M corresponds to the adduct shown in Fig. 2B (C_31_H_44_O_6_, monoisotopic mass 512.3 Da). [MNa]^+^ was observed as the base peak. This is in agreement with the experimental conditions used for the sample preparation (matrix system: dithranol/NaTFA). The insets in Fig. 4A,a and b show a comparison of measured and calculated accurate masses. The experimental result of *m*/*z*_exp_ = 535.3016 Da closely corresponds to the calculated mass of the monoisotopic peak of [MNa]^+^ (C_31_H_44_O_6_Na, *m*/*z*_calc_ = 535.3036 Da). Thus, the MALDI-TOF mass spectrum confirms the results obtained by NMR. DI-EI analysis generated the mass spectrum shown in Fig. 4B. The peak observed at *m*/*z* = 512.3157 Da (Fig. 4B,b) is interpreted as the monoisotopic peak of molecular ion [M]^+^ (C_31_H_44_O_6_: *m*/*z*_calc_ = 512.3138 Da, compare inset of Fig. 4B,a), in line with the structure shown in Fig. 2B. Various other fragment ions observed in the EI spectrum correspond to the anticipated structure, e.g. *m*/*z* = 494.3053 Da can be assigned to [M − H_2_O]^+^ (C_31_H_42_O_5_, *m*/*z*_calc_ = 494.3032 Da) and the signal at *m*/*z* = 107.0 Da corresponds to [CH_2_PhOH]^+^ (C_7_H_7_O, *m*/*z*_calc_ = 107.0 Da).

### 3.5 Adduct formation preserves BMVEC function

**Fig. 5:**
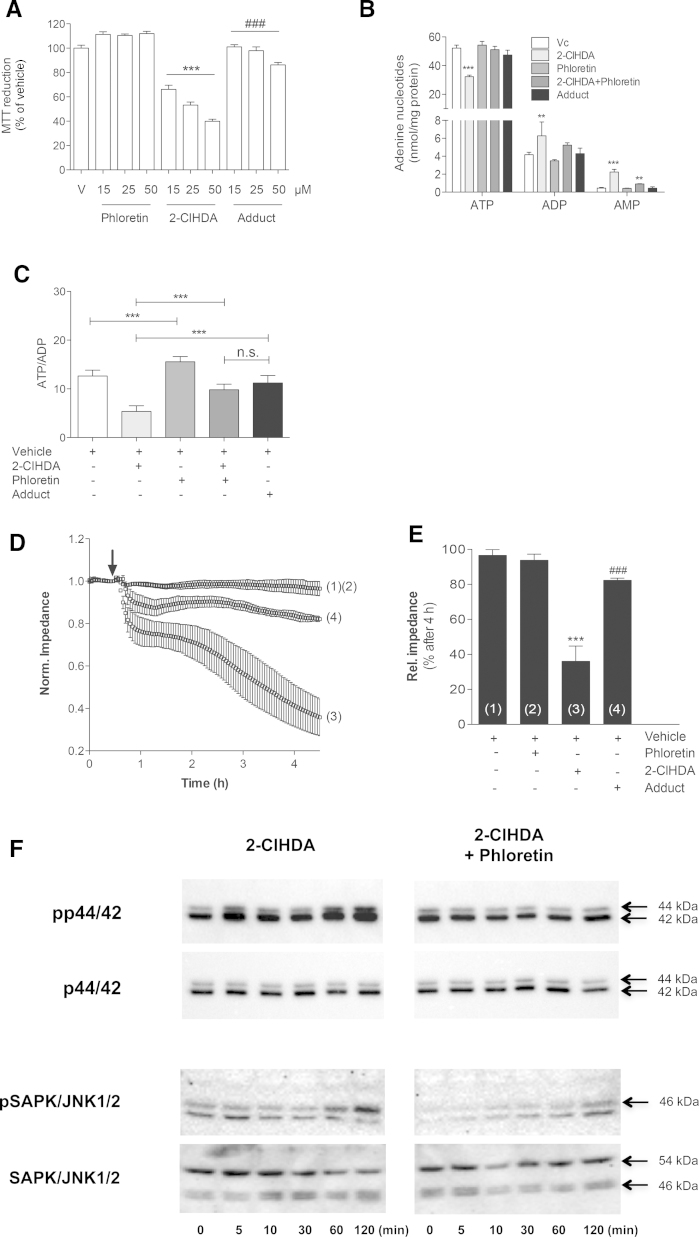
**Phloretin ameliorates cytotoxic properties of 2-ClHDA on brain endothelial cells.** (A) Results for MTT reduction in the presence of the indicated compounds are expressed as % of vehicle control (‘V’; DMSO, 0.4%) and represent mean ± SD from seven independent determinations. *** *p* < 0.001 vs. vehicle treatment; ^###^*p* < 0.001 vs. equal 2-ClHDA concentrations; one-way ANOVA and Bonferroni comparison). (B) Cellular adenine nucleotide levels (ATP, ADP, and AMP) were analyzed in the presence of the indicated compounds (25 μM) by HPLC. Vc = vehicle control. Results represent mean ± SD from six dishes. *** *p* < 0.001 vs. vehicle treatment, one-way ANOVA and Bonferroni comparison. (C) ATP/ADP ratios in the presence of the indicated compounds (25 μM). *** *p* < 0.001 vs. vehicle treatment, one-way ANOVA and Bonferroni comparison; n.s. = not significant. (D) BMVEC were plated on gold microelectrodes and cultured to confluence. Impedance of hydrocortisone-induced monolayers (7.5 × 10^4^ cells) was continuously monitored at 4 kHz. After monolayer stabilization, cells were incubated with vehicle (DMSO, 0.2%; ‘1’), phloretin (15 μM; ‘2’), 2-ClHDA (15 μM; ‘3’), or purified 2-ClHDA-phloretin adduct (15 μM; ‘4’). Impedance was monitored over 4 h. The arrow indicates addition of compounds. (E). Statistical evaluation of relative impedance values (1–4) after 4 h from (D). Impedance values were normalized to treatment start and represent mean values ± SD of four independent experiments (*** *p* < 0.001 vs. vehicle treatment; ^###^*p* < 0.001 vs. 2-ClHDA; one-way ANOVA and Bonferroni comparison). (F) BMVEC were incubated with 2-ClHDA (15 μM) or 2-ClHDA plus phloretin (15 μM each). Aliquots of cell lysates (50 μg protein/lane) were subjected to SDS-PAGE and transferred to PVDF membranes. Pan- or phospho-specific polyclonal antibodies against p44/42 or SAPK1/JNK1/2 were used as primary antibodies. Immunoreactive bands were visualized with HRP-conjugated secondary antibodies using the Bio Rad ChemiDoc system.

Next we investigated the impact of 2-ClHDA, phloretin, and the 2-ClHDA-phloretin adduct on metabolic activity of BMVEC. As expected and addressed earlier [19], MTT reduction was significantly attenuated by 2-ClHDA (Fig. 5A; 60% reduction at 50 μM) indicating significantly reduced cellular metabolic activity. The presence of phloretin tended to increase MTT reduction. Of note, the isolated 2-ClHDA-phloretin adduct had no effect on MTT reduction at 15 and 25 μM while a slight (14%) decrease was observed at 50 μM.

Comparable observations were made for the intracellular adenine nucleotide status: 2-ClHDA significantly reduced cellular ATP concentrations (from 52.2 to 32.2 nmol/mg protein vehicle vs. 2-ClHDA); this effect was almost completely attenuated in the presence of 2-ClHDA plus phloretin (51.1 nmol/mg protein) and the 2-ClHDA-phloretin adduct (47.5 nmol/mg protein; Fig. 5B). AMP levels were increased by 4.4-fold in 2-ClHDA-treated cells (2.2 vs. 0.5 nmol AMP/mg protein, 2-ClHDA vs. vehicle). Of note, addition of the adduct was without effect on the cellular AMP content, while in 2-ClHDA plus phloretin-treated cells AMP concentrations were 2-fold higher when compared to adduct-treated BMVEC. The differences were less pronounced in terms of ADP concentrations (6.3 vs. 4.2 nmol/mg protein, 2-ClHDA vs. vehicle). However, also ADP levels were higher in 2-ClHDA plus phloretin as compared to adduct-treated cells (5.2 vs. 4.2 nmol/mg protein). These findings were also reflected in the ATP/ADP ratios (Fig. 5C), although the difference between 2-ClHDA plus phloretin and adduct-treated cells did not reach statistical significance (*p* = 0.0619). Phloretin alone slightly increased the ATP/ADP ratio.

As reported earlier [10] and observed here, 2-ClHDA induces severe loss of BMVEC barrier function as revealed by ECIS measurements (Fig. 5D). In contrast, the covalent 2-ClHDA-phloretin adduct was almost without effect on barrier function. Statistical evaluation of the relative impedance data at 4 h is shown in Fig. 5E. These findings indicate that phloretin efficiently reduces deleterious effects of 2-ClHDA on metabolic and barrier function of BMVEC.

MAPK signaling plays a critical role in the regulation of barrier integrity [29,30]. In line, our earlier studies demonstrated that pharmacological antagonism of p44/42 and SAPK/JNK1/2 activation prevented 2-ClHDA-mediated BMVEC barrier dysfunction [19]. To investigate whether phloretin could interact with these events cells were incubated with 2-ClHDA in the absence or presence of phloretin. These experiments corroborated that phloretin led to substantially reduced activation of p44/42 and SAPK/JNK1/2 by 2-ClHDA (Fig. 5F).

To explore if these observations are accompanied by in vitro adduct formation, 2-ClHDA and phloretin were added to the supernatant of confluent BMVEC monolayers. Since 2-ClHDA may be converted to the corresponding acid and alcohol under these conditions [19], we have chosen short incubation times to (at least partially) overcome this limitation. Data from these experiments (Table 2) indicate that 19 (15 min) and 14% (60 min) of 2-ClHDA are recovered as adduct from the cellular supernatant. Approx. 5% of 2-ClHDA were recovered as cell-associated adduct (Table 2).

### 3.6 In vivo brain uptake of phloretin

**Fig. 6:**
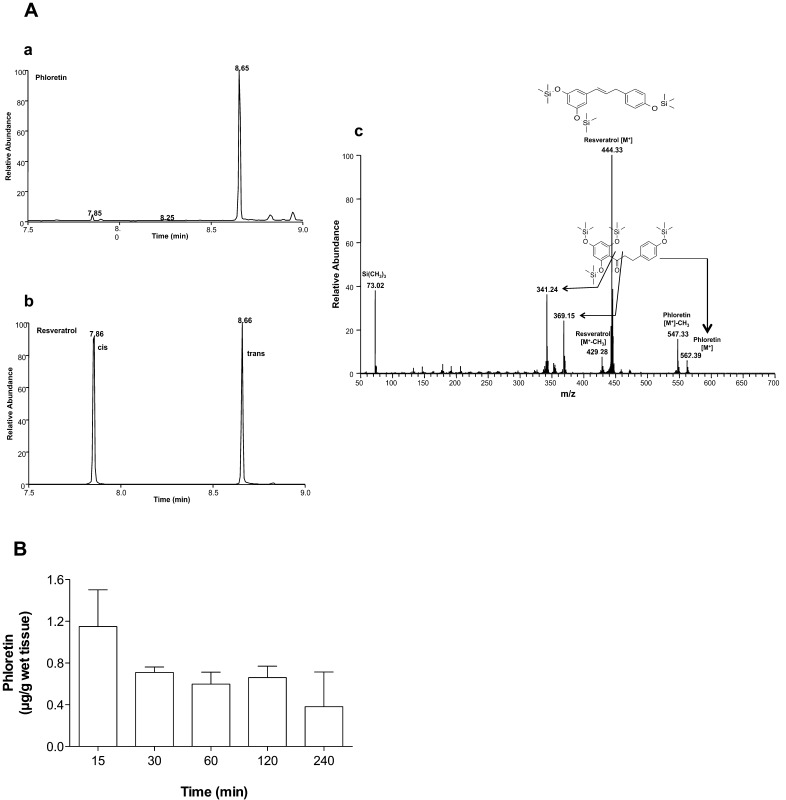
**Phloretin accumulation in murine brain after i.p. administration.** C57BL/6J mice (*n* = 3/group) received a single injection (i.p.) of phloretin (0.1 mg/g body weight). At the indicated times brains were removed, homogenized, extracted, and phloretin was analyzed by EI–GC–MS analysis in the SIM mode and quantitated using resveratrol as internal standard. (A) SIM trace of a representative brain lipid sample (15 min post phloretin application) containing resveratrol as internal standard. Phloretin (a) was monitored at *m*/*z* 562 [M^+^] and 547 [M^+^ − CH_3_], resveratrol (b) was monitored at *m*/*z* 444.3 [M^+^]. Note coelution of trans-resveratrol with phloretin. Fragment ion intensity ratios of phloretin and the internal standard are shown in (c). Mass assignment for the major fragments is indicated. (B) Time-dependent quantitation of phloretin in mouse brain homogenates after i.p. application. Results shown represent mean ± SD from three animals per time point.

To get an indication whether peripherally administered phloretin is able to cross the BBB in vivo C57BL/6J mice were injected i.p. with phloretin. At the time points indicated animals were anesthetized and transcardially perfused. Brains were removed, weighed, homogenized, extracted, and the corresponding phloretin TMS-ether derivatives were quantitated by EI–GC–MS. Fig. 6A,a shows SIM traces of TMS-ether derivatives of phloretin (analyzed from brain lipid extracts prepared 15 min post i.p. injection; diagnostic ion at *m*/*z* = 547, resulting from M^+^ − CH_3_) and resveratrol (diagnostic ion at *m*/*z* = 444; [M^+^]) which was used as internal standard. As is evident from the SIM trace, trans-resveratrol and phloretin co-eluted, while *cis*-resveratrol was separated from phloretin (retention time = 7.86 min; Fig. 6A,b). Fragment ion intensity (and mass assignment for the major fragments) ratios of phloretin and the internal standard are shown in Fig. 6A,c. Time-dependent accumulation of phloretin in mouse brain is shown in Fig. 6B. These experiments revealed maximum phloretin concentrations 15 min post application (1.2 μg/g brain tissue; based on the average mouse brain weight of 400 mg this corresponds to approx. 0.015% of totally administered phloretin). In brain of control animals phloretin was undetectable. These data indicate that phloretin is subject to transport across the murine BBB in vivo.

## 4 Discussion

Here we describe a phloretin-mediated detoxification pathway for a chlorinated fatty aldehyde that has detrimental effects on BBB function in vitro and in vivo. Members of the polyphenol family were reported to beneficially interfere with chronic and acute neurodegenerative conditions [31–33] that are invariably accompanied by different stages of oxidative stress [34]. Polyphenols are able to inhibit the formation of reactive species [35], reduce transition metal availability [36], and covalently bind reactive unsaturated aldehydes [37]. Systemic inflammation results in immune cell recruitment to the cerebrovasculature where neutrophils release MPO to generate MPO-derived oxidants [10]. During the present study we investigated the potential of phloretin, a dihydrochalcone, to act as a sink for the reactive chlorinated aldehyde 2-ClHDA. 2-ClHDA has potent signaling [11,38,39] and pro-apoptotic properties [10,19] and mimics some of the detrimental effects that are linked to plasmalogen deficiency in brains of Alzheimer's disease patients [40]. As reported here, adduct formation with phloretin occurs at ambient conditions via electrophilic aromatic substitution and subsequent cyclic hemiacetal formation. This process ameliorates most of the harmful effects of 2-ClHDA on brain endothelial cell function.

Initially we have determined adduct formation under different conditions (Fig. 1). These experiments revealed most efficient adduct formation in the presence of HDL particles. These findings could indicate that HDL (which is actively secreted by BMVEC; Ref. [28]) could provide a hydrophobic environment facilitating 2-ClHDA-phloretin interaction. Adduct formation is a relatively slow process with observed rate constants between 1.4 × 10^−2^ to 4.4 × 10^−3^ min^−1^ (Fig. 1). Nevertheless, phloretin (added in the presence of 2-ClHDA) provided protection against ATP depletion (Fig. 5B) and rescued barrier dysfunction [19] at 2-ClHDA concentrations comparable to what was reported for in vivo systems (1–20 μM; Refs. [9,41,42]). These observations could be due to the fact that adduct formation reduces 2-ClHDA concentrations to a sub-lethal range or suppresses early electrophile-mediated signaling events (i.e. p44/42 and SAPK/JNK1/2 activation; Fig. 5F) that are able to induce cell damage in a hierarchical manner. Our ongoing studies indicate that 2-ClHDA is able to covalently modify BMVEC proteins (C.N., N.K., E.M., and W.S.; unpublished). Adduct formation with phloretin would thus reduce the concentration of 2-ClHDA that is available to inflict alkylation damage (as reported for 4-hydroxynonenal, another reactive aldehyde formed in vivo; Ref. [43]) to cellular proteins.

NMR, enthalpy calculation, and HRMS (Figs. 2–4) suggest that adduct formation between 2-ClHDA and phloretin at ambient conditions (37 °C, pH 7.4) proceeds via aromatic electrophilic substitution followed by hemiacetal formation at the A-ring of phloretin, while the B-ring is apparently not involved. Formation of the transition complex starts with electrophilic attack of 2-ClHDA at C-5 of the A-ring. Subsequently, nucleophilic attack at the terminal aldehyde by a nearby hydroxyl group at C-6 leads to the formation of a cyclic hemiacetal as the stable end product. Whether metal catalysis contributes to adduct formation was experimentally not addressed during the present study. There is ample evidence that phloretin can interact at several stages of ‘oxidative stress’: Phloretin acts as antioxidant by scavenging radical species [44], inhibits the formation of lipid hydroperoxides [45], and covalently traps α,β-unsaturated aldehydes [37]. Zhu and colleagues [18] have reported that among several different polyphenol classes only flavan-3-ols, theaflavins, cyanomaclurins, and dihydrochalcones (all containing a phloroglucinol moiety, the A-ring of phloretin) are able to trap reactive carbonyl species. Among the active compounds, phloretin most efficiently binds acrolein and 4-hydroxynonenal. LC–MS and NMR analyses revealed that both, 4-hydroxynonenal and acrolein are subject to aromatic electrophilic substitution at the A-ring of phloretin followed by hemiacetal formation in aqueous solution [18]. Of note, acrolein can be generated by MPO-mediated oxidation of threonine and subsequent water abstraction of the 2-hydroxypropanal intermediate [46]. Involvement of the A-ring of phloretin was also demonstrated to be involved in scavenging of acetaldehyde and glyoxylic acid [47] as well as glyoxal and methylglyoxal [48]. Thus the reaction mechanism identified here for a chlorinated fatty aldehyde (Fig. 3) is in line with reports for other reactive carbonyl species. In terms of pharmacological applicability it should be noted that phloretin inhibits glucose uptake into brain and brain endothelial cells in vitro [49] and in vivo [50,51].

As noted above, the pharmacophore facilitating covalent trapping of reactive carbonyl species is the phloroglucinol moiety. Based on this structural element, LoPachin and colleagues [52] have demonstrated that simple β-dicarbonyl enolates like e.g. acetylacetone, 1,3-cyclopentandione, or 2-acetylcyclopentanone are able to provide neuroprotection from aldehyde-induced toxicity and thiol loss in vitro. The same group [53] reported that 2-acetylcyclopentanone protects against acetaminophen-mediated hepatotoxicity in vivo.

To fulfill a neuromodulatory role, polyphenols have to cross the BBB. Flavonoid uptake has been correlated with increased cognitive performance in experimental animal models, suggesting a neuroprotective role of these compounds [31,32]. Under our experimental conditions approx. 0.015% of phloretin (applied i.p.) was recovered in the brain lipid fraction of C57BL/6J mice (Fig. 6B) indicating penetration of the murine BBB. This might result from the fact that the logP of phloretin is >2 [27] since BBB penetration is optimal when the logP values are in the range of 1.5-2.7 [54]. In line, naringenin, a compound structurally related to phloretin (logP = 1.9; Ref. [55]) was shown to cross the BBB in vitro and in vivo [56]. Findings of the present study indicate that concentrations of phloretin (15 min post administration) in murine brain are approx. 6 μM (dropping to 2.2 μM at 240 min). This is in the range of cerebral concentrations of the neuroprotective antioxidant α-tocopherol (4 μM; Ref. [57]). Hypothetically these phloretin concentrations would suffice to scavenge the peak levels of 2-ClHDA that is generated under neuroinflammatory conditions in the murine brain (*C*_max_ ≈ 10 μM; Ref. [9]). However, in light of the rate constants determined for adduct formation (5.9 × 10^−3^ and 1.4 × 10^−2^ min^−1^, liposomes and HDL, respectively) the ability of phloretin to scavenge 2-ClHDA efficiently under in vivo conditions remains to be demonstrated.

In conclusion, we have characterized the reaction mechanisms governing covalent adduct formation between phloretin and 2-ClHDA, a process ameliorating detrimental effects of this chlorinated fatty aldehyde on brain endothelial cell function. Whether these findings can be translated into therapeutic applications to improve the outcome of neuroinflammatory conditions remains to be elucidated.

## Figures and Tables

**Table 1:** NMR data of the adduct corresponding to 3-(4-hydroxyphenyl)-1-(2,4,6-trihydroxy-3-tetradecyl-2,3-dihydrobenzofuran-7-yl)propan-1-one acquired in DMSO-d_6_. Numbering and structure is shown in Fig. 2B.

Position	δ(^1^H) [ppm]	Multiplicity	δ(^13^C) [ppm]
1	–	–	202.9
2	2.78	t	29.6
3	3.18	t	44.9
1′	–	-	131.5
2′, 6′	7.03	d	129.4
3′, 5′	6.66	d	115.2
4′	9.18	s	155.5
2″	5.73	d	107.8
2″ (OH)	7.53	d	–
3″	2.94	d	46.7
3a″	–	-	107.1
4″	10.66	s	160.9
5″	5.84	s	95.6
6″	13.17	s	164.8
7″	–	–	100.6
7a″	–	–	161.1
i	1.68	m	31.7
ii–xii	1.25	m	31.2
xiii	1.25	m	22.4
xiv	0.83	t	14.3

**Table 2:** 2-ClHDA-phloretin adduct formation in BMVEC cultures. Confluent BMVEC monolayers were preincubated in glucose-containing HBSS (2 ml) in the presence of phloretin for 60 min. After addition of 2-ClHDA (25 μM) the cellular supernatant and cell monolayers were extracted after 15 and 60 min and analyzed either by HPLC–UV detection (2-ClHDA-conjugated phloretin) or by NICI–GC–MS (2-ClHDA).

Time (min)	Supernatant^a^		Cell-associated^a^		Total^b^
	2-ClHDA	Adduct	2-ClHDA	Adduct	
15	74 ± 9.7^c^	19 ± 3.4	14 ± 4.6	5 ± 0.8	112
60	41 ± 11.9	14 ± 2.6	24 ± 1.6	4 ± 0.3	83

a Results are given as % of added 2-ClHDA (25 μM).

b Sum of analytes recovered from the medium and in cell association.

c Results shown represent mean ± SD (*n* = 3).
