# Supplementary figures and images for: Covalent adduct formation between the plasmalogen-derived modification product 2-chlorohexadecanal and phloretin
Source: Biochem Pharmacol. 2015 Feb 15;93(4):470–81. doi: 10.1016/j.bcp.2014.12.017 (PMC4321883; doi:10.1016/j.bcp.2014.12.017)

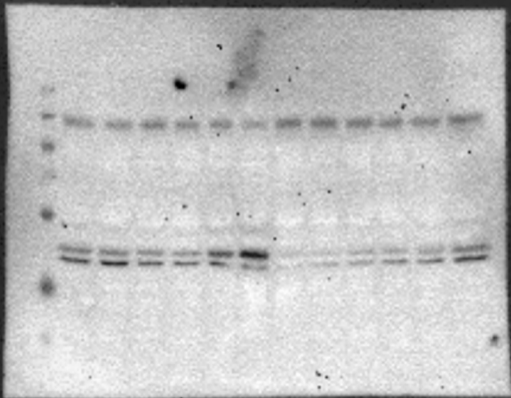

Supplement: Supplementary file 1 [file mmc1.pdf]
